# Advances in Reproductive Endocrinology and Neuroendocrine Research Using Catfish Models

**DOI:** 10.3390/cells10112807

**Published:** 2021-10-20

**Authors:** Balasubramanian Senthilkumaran, Sonika Kar

**Affiliations:** Department of Animal Biology, School of Life Sciences, University of Hyderabad, P.O. Central University, Hyderabad 500046, Telangana, India; 17laph06@uohyd.ac.in

**Keywords:** catfish, sex differentiation, gonadal development, gametogenesis, neuroendocrine regulation

## Abstract

Catfishes, belonging to the order siluriformes, represent one of the largest groups of freshwater fishes with more than 4000 species and almost 12% of teleostean population. Due to their worldwide distribution and diversity, catfishes are interesting models for ecologists and evolutionary biologists. Incidentally, catfish emerged as an excellent animal model for aquaculture research because of economic importance, availability, disease resistance, adaptability to artificial spawning, handling, culture, high fecundity, hatchability, hypoxia tolerance and their ability to acclimate to laboratory conditions. Reproductive system in catfish is orchestrated by complex network of nervous, endocrine system and environmental factors during gonadal growth as well as recrudescence. Lot of new information on the molecular mechanism of gonadal development have been obtained over several decades which are evident from significant number of scientific publications pertaining to reproductive biology and neuroendocrine research in catfish. This review aims to synthesize key findings and compile highly relevant aspects on how catfish can offer insight into fundamental mechanisms of all the areas of reproduction and its neuroendocrine regulation, from gametogenesis to spawning including seasonal reproductive cycle. In addition, the state-of-knowledge surrounding gonadal development and neuroendocrine control of gonadal sex differentiation in catfish are comprehensively summarized in comparison with other fish models.

## 1. Introduction

Catfish (order Siluriformes) are diverse groups of ray-finned fish that are mostly benthic or bottom dwellers [1] and are named so for their prominent barbells that resembles a cat’s whiskers. They represent one of the largest groups of freshwater fishes. They are scaleless and are defined by features of the skull, spine in front of their fins and swim bladder. Catfish have widely been caught and farmed for food, due to high protein content, for hundreds of years across many continents. In addition, some species are also reared as ornamental fish or research animals due to more adaptability for artificial spawning and culture. Several air breathing catfish (family- Clariidae) consisting of about 48 species [2] together with Heteropneustidae and shark catfish (Pangasiidae) species are widely cultured in the Asia and the Africa due to relatively higher fecundity, high tolerance to hypoxia, etc. Some of the other widely cultured species includes channel catfish, *Ictalurus punctatus* and blue catfish, *I. furcatus*. Additionally, genus *Kryptopterus* contains various small and transparent catfishes described as glass catfish [3].

Catfishes also undergo a seasonal reproductive cycle characterized by distinct stages [preparatory, pre-spawning, spawning, post-spawning and resting] in subtropical countries including India controlled by a hormone regulatory pathway primarily involving gonadotropin-releasing hormone (GnRH), luteinizing hormone (LH), follicular stimulating hormone (FSH), growth hormone, melatonin, and sex steroid hormones [4,5]. Thereby, a gonadotropin (GTH) surge usually facilitates spontaneous oocytes maturation, ovulation or spermiation in nature. However, catfish usually do not spawn or spermiates in laboratory or culture conditions [6,7,8]. Apart from these, neuroendocrine factors such as, neurotransmitters and neuropeptides also play a crucial role in neuroendocrine control of gonadal development and maturation [9]. Testosterone (T) and 17β-estradiol (E_2_) exert a primary role in gonadal development locally, by several positive and negative feedback actions at the levels of brain and pituitary across endocrine axis [10]. Evidently, spawning strategies for catfish can be divided into two main categories: natural and artificial spawning wherein artificial spawning is performed by inducing females to ovulate with hormones, followed by which eggs are hand-stripped and fertilized in vitro.

As endocrine system regulates gonadal development, growth, and reproduction, hence, fish endocrinology has been the focus of various studies for basic understanding of these physiological events and for advances in aquaculture. Over the decades, many fish species have been used to study various aspects of endocrinology in vivo. Several genome editing and transgenesis studies have also been done to understand the complexity of endocrine functions and regulation in fish. This review summarizes the present knowledge and key evidence on catfish being used as research models for studying fish endocrinology. To begin with, key evidence of neuroendocrine control of gonadal development and sex determination/differentiation are discussed followed by understanding of steroidogenic regulation in catfish. Key findings on how catfish models have been used to understand gene regulation and function using gene knock out/transient gene knock down through short interfering RNA (siRNA) are listed. Furthermore, wherever necessary the research findings from catfish models were compared with other teleostean counterparts for comprehensive review of literature.

## 2. Neuroendocrine Regulation-GnRHs

Teleost fish are excellent models to study neuroendocrine control of reproduction. Fishes synthesize LH and FSH from anterior pituitary under the control of hypothalamus GnRHs to regulate early gametogenesis, steroidogenesis and ovulation/spermiation. Hence, puberty is governed by GnRH and certain gonadal steroids. GnRH release is controlled by several neurotransmitters and neuropeptides. Multiple forms of GnRH have been identified and localized in brains of most of the non-mammalian vertebrates, including, fish [11,12,13]. In the African catfish, *C. gariepinus*, two genomic isoforms of GnRH have been characterized till date [11] along with two forms of GnRH receptors with varied tissue distribution but no differences in ligand selectivity [14]. The first teleostean GnRH receptor was isolated from the African catfish [15]. Since the discovery of GnRH in vertebrates over three decades, considerable progress has been made towards understanding of the neuroendocrine control of gonadal development and reproduction in mammals and fish which has been reviewed extensively by Zohar et al. [16]. Molecular cloning/characterization of GnRH2 precursor cDNA and its regulation by ovarian steroids were demonstrated in the stinging catfish, *Heteropneustes fossilis* [17]. Furthermore, the stimulatory and inhibitory interactions between GnRH- neuropeptides, including neuropeptide Y (NPY) and GnRH- neurotransmitters, including DA and γ-aminobutyric acid (GABA) has been reviewed and demonstrated well by Trudeau [10] using goldfish model. The effects of 5-hydroxytryptamine (5-HT), GABA and NPY on in vitro release of GnRH have been well demonstrated in a perciform fish [18]. In addition to this, the functional significance of GnRH–kisspeptin (a neuropeptide encoded by the *kiss* gene, the “gatekeeper” of puberty) in teleostean reproduction and their associated receptors have been reviewed by Gopurappilly et al. [19] including various catfish models. After identification of *kiss2* and *GnRH2* in the stinging catfish, *H. fossilis*, [17,20], a recent study demonstrated that *kiss2*-*GnRH2* signaling is involved in photo-thermal-mediated mechanisms controlling reproduction in catfish [21]. Evolution of *kiss* functions in teleost along with the common regulatory mechanism of hypothalamo-hypophyseal gonadal (HHG) axis has been also reviewed by Kanda [22]. Taken together, these complex systems stimulate gametogenesis and sexual behaviors through the activation of HHG axis in teleosts including catfish.

In addition to HHG axis, endocrine feedback system at thyroid axis also contributes to homeostasis maintenance, growth, differentiation, and reproduction in teleosts including catfish [23,24]. Hence, thyroid hormone (TH) also plays a critical role in brain development/function. THs are also known to modulate reproductive system during different developmental stages in fish [25] and several catfish models have been extensively used over the decades to decode the underlying mechanisms of endocrine control of reproduction and to identify various markers associated functionally across the endocrine axes.

### 2.1. GTH Duality

GTH, a glycoprotein hormone, stimulates gonadal maturation and development in most of the vertebrates. In many teleosts, including salmonids and rainbow trout, two types of GTHs, GTH-I (FSH- like) and GTH-II (LH- like) have been characterized [26,27,28,29,30] which are equipotent in stimulating E_2_ production, hence, stimulating steroid synthesis, although localized in separate cells. However, in primitive teleosts such as eel [31,32] and catfish [33,34,35], only a single GTH (GTH-II) has been characterized which is known to regulate the entire process of gonadal development. The possibilities implicating about the absence of FSH-like GTH-I in catfish has been attributed by Joy [36]. The African catfish FSH-R responded clearly to the highly purified African catfish LH when expressed in a mammalian cell line [37] and the channel catfish FSH-R responded to human chorionic gonadotropin (hCG) although the response was weaker than when challenged with human FSH [38,39].

Furthermore, GnRH’s role in the stimulation of LH synthesis in catfish has been reviewed by Schulz et al. [40]. In line with this, it has been reported that the pituitary gonadotrophs are known to be activated strongly during initiation of spermatogenesis in the African catfish, *Clarias gariepinus* [41].

In addition, seasonal cyclicity of GTH-II has been demonstrated in various catfish species with standardized protocols as well as comparison with nuclear E_2_ receptor binding [42,43]. However, since there is no distinction of GTH-I and GTH-II, it is referred as GTH-II or LH in these catfish species.

### 2.2. Neurotransmitters, Neuropeptides and GnRH-GTH Axis

Neurotransmitters such as, catecholamines (CA)- dopamine (DA), norepinephrine (NE), adrenaline (A) and serotonin (or 5-HT) are low molecular weight organic nitrogen compounds. In terms of synthesis, packaging, release, and degradation, the amine neurotransmitters fall somewhere between the properties of other small-molecule neurotransmitters and those of the neuropeptides. Neurotransmitters such as monoamines, amino acids and peptides are known to involve in the neuroendocrine control of reproduction.

#### 2.2.1. Serotonin

Serotonergic system plays a critical role in orchestrating HHG axis to promote gonadal growth in vertebrates including fish. Enzyme, tryptophan hydroxylase (*tph*), is a crucial rate-limiting enzyme for serotonin synthesis. Selective up regulation of *tph* expression and serotonin levels in brain has been shown in XY male tilapia and abolition of such a phenomenon leads to complete sex reversal during early development [44] which was evident by para-chlorophenylalanine (pCPA) (a *tph* blocker) treatment [45,46]. Such a phenomenon was also well demonstrated in catfish with gender differences where in pCPA skewed the population towards females by initiating ovarian differentiation [47]. A single injection of pCPA decreased the content and activity of serotonin in *Channa punctatus* [48]. Similarly, pCPA reduced hypothalamic serotonin level and impaired GnRH and LH secretion in the Atlantic croaker [49]. Furthermore, in fish, serotonergic system can be modulated by a variety of chemical substances and environmental factors. For example, diurnal variations in serotonin content and turnover in response to melatonin have been demonstrated in *C. punctatus* [50] and *H. fossilis* [51,52]. In teleost, serotonin receptors have been identified and characterized in several species in peripheral as well as gonadal tissues, as reviewed by Prasad et al. [53]. Furthermore, high hypothalamic monoamine oxidase (MAO) activity with a relatively high turnover of serotonin has been observed during recrudescence in catfish, relating to high temperature and breeding activity [54,55]. In addition, the involvement of serotonin and MAO has been well demonstrated in feedback regulation of E_2_ in catfish [52,56,57,58]. The half-life analysis and turnover of MAO (using pargyline) were conducted to reveal its involvement in E_2_-modulated feedback regulation of GnRH-GTH axis [58]. Ovariectomy-induced changes in plasma levels of GTH partly mediated by MAO activity and E_2_ feedback action on serotonin metabolism were also observed in a seasonal-dependent manner [56,57,58]. The role of serotonin in fish reproduction including studies in catfish except a few recent reports [59,60] has been extensively reviewed by Prasad et al. [53].

#### 2.2.2. CAs

CA, an important component of monoaminergic system in the hypothalamus, modulates the levels of GnRH with subsequent release GTHs in teleosts including catfish [57,61,62]. The CAs include L-DOPA, DA and NA, all of which plays decisive roles in various physiological processes to control reproduction. In the African catfish, dopamine acts as an endogenous inhibitor of GnRH-stimulated GTH release during spermatogenesis and vitellogenesis [63,64].

Among the CAs, DA exerts an inhibitory control on GTH while NA stimulates GTH by regulating GnRH synthesis in teleost [65,66]. Additionally, negative feedback by sex steroids also involves in activation of inhibitory DA system [10]. In the Indian stinging catfish, high temperature decreases DA activity and increases NA activity, which is a stimulatory signal for GTH-II [57]. Mamta and Senthilkumaran [67] demonstrated *gfrα-1* plausibly entrains GnRH-GTH either directly or indirectly, by partially targeting CA-ergic activity. In addition, another study in catfish demonstrated catecholestrogens (CE) related enzymatic changes in during GnRH analogue-induced ovulation and suggesting E_2_ modulation of catechol-*O*-methyltransferase (COMT) activity [68]. Ovariectomy and/or E_2_ replacement also modulated hypothalamic COMT activity in catfish. In addition, season-specific changes in hypothalamic COMT demonstrated its involvement in CA/CE mediated control of GTH [69]. Enzyme tyrosine hydroxylase (*th*) regulates the levels of GnRH in brain and GTHs in the pituitary. In *H. fossilis* brain, *th* activity and its correlation with the annual reproductive cycle [70] is well demonstrated and is known to be modulated by cyclic AMP- protein kinase A and protein kinase C [71]. Furthermore, sex-specific differential expression of *th* was observed in early developmental stages in male and female catfish brain that correlates with CAs [62]. Furthermore, a study in the Indian catfish demonstrated sexual dimorphism in *th*-positive neurons in the preoptic area of the brain [72]. In some air breathing catfish species, coexisting in sub-tropical waters, there is seasonality in the dominance of the CA during the reproductive cycle wherein DA content and turnover were found to be high during the resting phase and decreased as breeding season progressed with a concomitant increase in NE turnover [57] unlike goldfish wherein the DA inhibitory tone is high. The turnover studies were explicitly performed using α-MPT to depict content and turnover of CA in catfish. Furthermore, NE was high in pre-spawning phase and A was high in spawning phase but not in resting phase. In line with this, administration of a single high dose of GnRH analogue facilitated induced-spawning and the periovulatory changes of monoaminergic system has been well demonstrated for the first time in catfish. Furthermore, precise action of CA on GTH- release has been well studied using specific blockers/precursors in ovariectomized catfish [57,66,69]. Overall, photoperiod, temperature, and E_2_-negative feedback act on CA to regulate GTH secretion.

#### 2.2.3. GABA

GABA is an important amino acid neurotransmitter. Studies in teleost, including goldfish, rainbow trout and catfish, had confirmed the presence of the metabolic enzymes of GABA in fish brain [73,74,75,76]. A pioneering investigation partially characterized the GABA receptor [77] followed by the demonstration of an uptake system in the brain of channel catfish [78]. In teleosts, including the Indian catfish, GABA is known to stimulate GTH-II release during puberty (independent of the DA system) and its distribution in catfish forebrain showed seasonal variation which could be altered negatively upon ovariectomy and restored upon E_2_ replacement [65,79]. A recent study in catfish demonstrated the role of laser puncture exposure on gonad maturation by examining GABA release in the brain [80].

#### 2.2.4. Neuropeptide Y

NPY, a 36 amino-acid neuropeptide, is involved in various physiological and homeostatic processes including stimulation of appetite. NPY has been indentified and demonstrated in several fish species including the *I. punctatus*, *C. batrachus* and *C. gariepinus* [81,82,83,84,85]. Increase in NPY during fasting is consistent with results in mammals [86] and fish models, including channel catfish [87]. Significance of NPY in the regulation of GnRH–LH axis was demonstrated by Subhedar et al. [88] using *C. batrachus*, also known as *C. magur*. Involvement of NPY and NPYY1 receptors was evident in regulation of GnRH–LH complex and GH cells in catfish pituitary [82,83]. However, all these studies showed localization pattern of NPY using heterologus antiserum. It is important to use homologous system to delineate the localization pattern precisely. In line with this, Sudhakumari et al. [85] precisely localized NPY transcript and protein in the preoptic area of the brain in *C. gariepinus* using homologous system. In addition, the authors demonstrated higher expression of NPY in the brain during pre-spawning phase as compared to other reproductive phases. Transient silencing of NPY-esiRNA (directly into the brain) decreased the expression of *tph2*, *cfGnRH*, *th*, *hsd3b* in brain and LH-b/GTH-II in pituitary in addition to several ovary-related transcripts indicating NPY’s role in ovarian development through GnRH-GTH axis. Thus, the authors established possible interaction of NPY with GnRH-GTH axis.

### 2.3. Brain Sex Differentiation/Dimorphism

Studies on pubertal development have been conducted in various fish species including catfish [35,89,90] suggesting that sex steroids regulate the development of the HHG axis in teleost. Furthermore, its correlation with testicular function has been reviewed by Blázquez and Trudeau [91]. Gonadectomy during later stages of gonadal recrudescence increases LH secretion in several teleost including the African catfish and the Indian catfish which can be restored by treatment with testosterone/E_2_ [66,92,93,94,95]. Ovarian aromatase, *cyp19a1a*, is known to be involved in conversion of androgens to estrogens and is also known for its role in sex reversal [96]. However, teleost also produce brain aromatase, encoded by *cyp19a1b* which synthesize high amounts of neuroestrogens [97] plausibly along with the action of its related transcription factors such as *ftzf1* and *foxl2* [98] as seen in catfish, leading to “Brain sex differentiation”. In teleost, most of the earlier reports tend to suggest that gonadal sex differentiation drives brain sex differentiation which has been reviewed extensively by Senthilkumaran et al. [99]. Nevertheless, the influence of brain serotonergic system on gonadal sex development in catfish is well demonstrated indicating the existence of “Brain sex differentiation” in teleosts including catfish. However, yet the brain sex changes are questioned as a “consequence” or “cause” to gonadal sex determination/differentiation [44,47,89].

**Figure 1:**
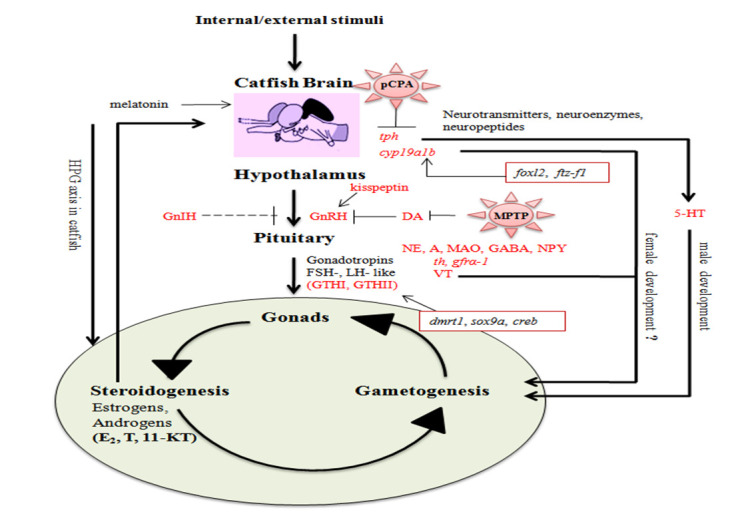
Schematic representation of neuroendocrine control of reproduction in catfish.

Additionally, teleost models including catfish have been used extensively to study neurotoxicity [100] and neuroendocrine disruption [101]. Neurotoxicity studies are important to identify promising neuroprotective agents for example, ascorbic acid for Al-induced neurotoxicity which was demonstrated using *C. gariepinus* [102]. In line with this, Mamta and Senthilkumaran [67] used 1-methyl-1,2,3,6-tetrahydropyridine (MPTP), to demonstrate the interaction of GDNF and DA-ergic system in catfish brain. In addition, controlled release of sex steroids using osmotic pump altered brain GnRH1 and CA-ergic system dimorphically in the African catfish providing insights into the reproductive toxicity of sex steroid analogues during gonadal recrudescence [103]. The schematic representation on neuroendocrine control of reproduction in catfish has been depicted in the Figure 1.

## 3. Gonad genesis

Gonad, in most fish species including catfishes, has bipotential fates to form ovary or testis depending upon a sex determination/differentiation cue [96,104,105] and various factors after which gonadal differentiation and further development of gonad takes place. Some hermaphrodite fishes can change their sex uni-directionally or bi-directionally during their life cycle, however, catfishes show gonochoristic pattern. Sex differentiation in fish is characterized by differential expression of related genes [106,107,108,109]. However, environmental cues, such as, temperature also plays a crucial role in sex differentiation in a few fish species including *C. gariepinus* [110] and *I. punctatus* [111]. Environmental sex determination in fish has been reviewed by Baroiller et al. [112].

### 3.1. Sex Determination/Differentiation, Gonadal Development and Growth

In mammals, the discovery of sex determining region Y, SRY gene, demonstrated its crucial role in testicular development [113,114]. However, the same has not been identified in fish except for a study involving identification of Y-chromosome specific molecular markers in a cyprinid fish using *sry*-specific PCR primers [115]. In fish, *dmy* or *dmrt1b* (duplicate copy of *dmrt1*) was found to be master sex determination gene, which was identified in the Japanese medaka, *Oryzias latipes* [116,117] as well as in *O. curvinotus* [118]. Following which, *dmrt1* have also been identified as testis-related gene in *Cynoglossus semilaevis* [119] and with multiple forms in catfish [120]. Thereafter, several studies were performed in various fish species including catfish to indentify crucial sex determination/differentiation genes [121] wherein several candidate genes for sex determination/differentiation were elucidated, for example, *amhy* in the Nile tilapia, *Oreochromisniloticus* [122], the Patagonian pejerrey, *Odontesthes hatcheri* [123] and *O. bonariensis* [124]; *amhr2* in *Takifugu rubripes* [125]; sdY in the rainbow trout, *Oncorhynchus mykiss* [126]; *gsdf* and *sox3* in *O. luzonensis* and *O. dancena* [127,128]. The cellular, molecular and physiological aspects of sex determination/differentiation in teleost have been reviewed by Sandra and Norma [129]. Furthermore, epigenetic characterization of sex chromosones were examined in two species of bullhead catfish (Amblycipitidae), *Liobagrus marginatus* and *L. styani* [130]. The genetic and epigenetic processes involved in regulation of sex-change in fish have been well reviewed by Ortega-Recalde et al. [131]. Additionally, sex determination/differentiation and feminization in the Southern catfish, *S. meridionalis* [132] and the channel catfish, *I. punctatus* [133] has been reviewed wherein *gdsf* and *cxcl12* plausibly initiated testicular differentiation as demonstrated in channel catfish. The genetic basis of sex determination/differentiation in fishes has been reviewed by Nagahama [134]. Several genes involved in sex determination/differentiation, such as *dmrt1*, *sox9*, *foxl2*, *bcar1* [135], and *cyp19* in catfish have been also identified. In addition to this, another study in catfish revealed the role of *ckit* in germ cell proliferation, development, and maturation during gonadal recrudescence [136]. Dimorphic expression of various transcription factors and steroidogenic enzyme genes has been demonstrated by Raghuveer et al. [47] during critical period of gonadal differentiation in catfish. Detailed analysis of various genes involved in sex differentiation in catfish has been reviewed [137].

### 3.2. Gonadal Recrudescence and Sex Reversal

Most of the fishes exhibit seasonal cycle in reproduction in the subtropical and tropical countries. The release of gametes from the body into the surrounding water is called spawning in fish. Some fishes are daily breeders (such as zebrafish) and some spawn during a specific season/period (seasonal/annual breeders like catfish) due to several environmental cues. During the breeding season of the species, the gonads attain full maturity followed by spawning. Gonadal recrudescence occurs after spawning subsequently to entrain seasonal/reproductive cycle. The breeding season and hence the spawning period is extremely variable among the bony fishes. Some seasonal breeders spawn only once (catfish), others twice (common carp), while still others may spawn several times during a year. Catfishes, generally, spawn annually during monsoon in the subtropical countries. Additionally, bony fishes can reverse their sex according to various environmental/social cues during their lifetime [96,138,139], however, it varies from species to species. Concepts and mechanisms involving sexual plasticity and gametogenesis in fishes including catfish has been covered extensively in “Sexual Plasticity and Gametogenesis in Fishes” by Senthilkumaran [140] and co-authors. Despite these, clear information about gonadal differentiation in sex-changing fishes remains limited. In catfish, female-to-male sex reversal has been achieved by fadrozole (aromatase inhibitor) and tamoxifen (estrogen receptor antagonist) treatment [141,142,143] as well as with pulsatile treatment of methyltestosterone (MT) and ethynyl estradiol (EE_2_), as demonstrated by Raghuveer and Senthilkumaran [120]. Furthermore, functional feminization of the channel catfish, *I. punctatus*, was demonstrated through treatment of estrogen diet [142,143]. Hence, estrogens, in teleost, are responsible for ovarian differentiation and feminization although the detailed mechanism involved remains elusive. However, potential androgens like 11-ketotestosterone (11-KT), MT and even non-aromatizable androgen [144] also produced female dominant populations in blue catfish and channel catfishes suggesting that no hormonal treatment could direct masculine sex determination [143,145]. Incidentally, treatment of MT occasionally resulted in intersex in catfish [120]. Hormonal induction of sex-reversal in fish including catfish has been extensively reviewed by Pandian and Kirankumar [146].

## 4. Gamete Maturation

Gonadal maturation is a critical event wherein gonads undergo cyclic morphological and physiological changes to produce functional gametes during the spawning phase with the help of several gene/factors and hormones. Artificial induction is used to advance the maturation of gonad in seasonal breeders (like catfish and eel) during the off-breeding season. This was first time demonstrated by Miura et al. [147] using the Japanese eel wherein, hCG injection could induce spermatogenesis. As hCG shares the same receptor as LH, studies were carried out to use hCG or ovaprim to advance gonadal development/maturation in teleost instead of GnRH analogues [148,149,150]. All these techniques have been adopted from the first discovery of ‘*LinPe*’ technique for induced breeding in fishes. This has been well established in several catfish [62,151]. In fact, controlled release of hCG via osmotic pump resulted in off-season breeding in catfish [150].

### 4.1. Final Oocyte Maturation

Final oocyte maturation (FOM), in fish, is promoted by the maturation inducing steroid, 17α,20β-dihydroxy-4-pregnen-3-one (17α,20β-DP) which is produced in ovarian granulosa cells by *hsb20b*, a key enzyme that initiates maturational events [152]. Furthermore, in teleost, shift in steroidogenesis from E_2_ to 17α, 20β-DP seems to be a crucial step during oocyte maturation [153,154]. Eventually, promoter motif analysis of *hsb20b* in catfish and rainbow trout demonstrated that *hsb20b* type B of rainbow trout had no promoter activity while *hsb20b* type A of rainbow trout and catfish *hsb20b* promoters showed basal promoter activity, wherein, cAMP responsive elements were the key regulators along with *crebs* [155] which was also indentified in the promoter motif of *cyp19a1a* [156,157]. Additionally, *cyp19a1a* expression is also crucial to understand the molecular mechanisms that precede ovarian differentiation/development. In vertebrates including teleost, *foxl2* is one of the earliest markers of ovarian differentiation. In addition, *ad4bp/sf-1*, *foxl2* and *ftz-f1* regulated *cyp19a1a*/*b* expression directly or indirectly in various fish species including catfish [97,156,158,159,160,161,162,163]. Furthermore, cAMP regulated *hsb20b* up-regulation in catfish [155]. A single form of *creb* was identified and characterized in *C. gariepinus* during FOM unlike multiple forms in the Nile tilapia, *O. niloticus* [164]. In this line, studies in common carp, suggested plausible roles for *ptx* and *thoc3* in ovarian growth, maturation/recrudescence upon functional analysis [165,166]. However, such an observation is yet to be investigated in any catfish species. Transcriptional interaction of Pax2 on *wnt5* also attributed to ovarian development in catfish explicitly [167] indicating multiple regulatory factors involved in gonadal function. Another report compared oocyte maturation of teleost with mammals to explicitly describe the phenomenon [168]. In fact, several of these studies in catfish were well complemented with enzyme activity assays to substantiate gene expression analysis authenticating downstream action [169].

Variety of hormones/metabolites/neurotransmitters showed oocytes maturation effects in addition to maturation-inducing hormone (MIH) in catfish species. This included cortisol, vasotocin (VT), CEs and CAs [61,170,171,172,173,174,175,176,177,178]. Both GTH and ovarian steroids modulate VT levels in catfish to influence follicular growth, ovulation, and spawning [174,175,179]. Incidentally, serotonin also induces oocyte maturation in fish and mollusks [180,181,182,183,184,185] which is yet to be explored in any catfish species. Despite these findings, MIH remains to be 17α,20β-DP in catfish too like some teleosts [176]. Catfish do not spawn in captivity without induction that may perhaps explain presence of various oocyte maturation inducing agents in vivo.

### 4.2. Sperm Maturation

In the African catfish, testicular development includes four stages that are distinguished by the presence of spermatogonia alone; spermatogonia and spermatocytes; spermatogonia, spermatocytes and spermatids; and finally, all germ cell stages, including spermatozoa [35,89]. In fish, GTHs show prominent steroidogenic potency at the onset of spermatogenesis and during rapid testicular growth and thier receptors have been localized in testicular tissue, also in the milt of channel catfish and in the seminal vesicles of the African catfish [37,38,39]. Maturation-inducing steroids such as 17α,20β-DP have been implicated in sperm maturation of teleosts to some extent including catfish [140,186]. Moreover, steroids T and 11-KT (a potent androgen in fishes) are responsible for sperm maturation and testicular development [186]. As described in the previous section, *dmrt1* along with other factors are known to be the molecular players in testicular differentiation and gamete maturation. In addition, several findings suggested that *wt1*, *ad4bp/sf-1*, *nr2c1*, *gata4*, *sox3*, *sox9*, *sycp3* and *pfpdz1* have a potential role in the testicular development, maintenance, and recrudescence in catfish by favoring spermatogenesis [187,188,189,190,191]. However, studies on transcriptional networks between *nr2c1* and other factors are necessary to demonstrate their interaction during testicular development and spermatogenesis.

## 5. Steroidogenic Enzyme Gene Regulation, Transcription Factors and Co-Modulators

Several genes/factors have been identified in teleost implicating their crucial roles in gametogenesis and gonadal steroidogenesis and most of which are regulated directly/indirectly by pituitary GTHs [137]. Steroidogenesis starts with rate-limiting transport of cholesterol into mitochondria [192] mediated by steroidogenic acute regulatory protein (StAR). StAR gene has been identified and characterized in teleosts, including rainbow trout, the African catfish and medaka [193,194,195]. Enzyme, *cyp11a1*, is involved in the conversion of cholesterol to pregnenolone, which thereby initiates the whole process of steroidogenesis including production of active steroids like 17α,20β-DP, T, 11-KT and E_2_ [168,196] via action of several steroidogenic enzymes genes which have been well identified and characterized in many teleost including catfish together with their associated transcription factors as evident from promoter motif analysis of the steroidogenic enzymes which has been reviewed in detail by Rajakumar and Senthilkumaran [169].

In addition to these, over a decade, next generation sequencing (NGS) techniques has been widely utilized for the identification of sex-related candidate genes and genetic markers using catfish models including red tail catfish [197]; the Hong Kong catfish [198]; amur catfish [199]; channel catfish [200,201]; yellow catfish [202,203,204,205] and the Indian and the African catfish [unpublished data] by investigating gonadal transcriptomes. These studies have provided a valuable genomic resource for further investigating the genetic basis of sex determination/differentiation and would aid in understanding more about sex-controlled breeding in catfish with a scope to extend this information to other teleost species.

## 6. Gene Knockout/Knockdown/siRNA Based Transient Gene Silencing

In the last few decades, there have been major advances in the field of gene/protein expression analysis to delineate their function in the organism. Many of the expression analysis techniques have been standardized in teleost including quantitative PCR, western blot, northern blotting, reporter assays, and high-throughput techniques like RNAseq and microarrays together with localization techniques such as in situ hybridization for mRNA and, immunohistochemisty/cytochemistry and immunofluroscence for protein.

**Figure 2:**
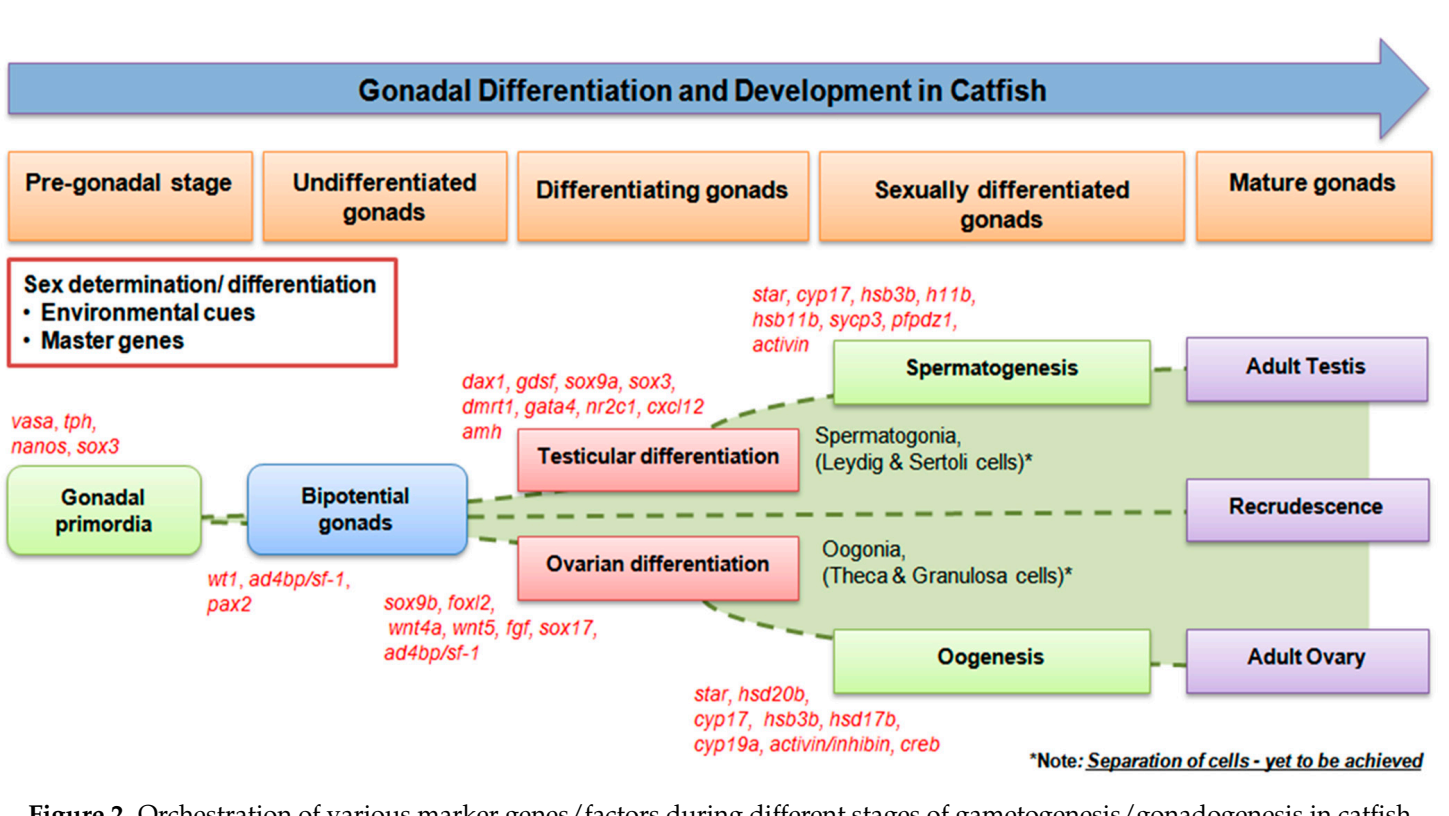
Orchestration of various marker genes/factors during different stages of gametogenesis/gonadogenesis in catfish.

However, in recent years, the field of reverse genetics has been evolving widely with the development of novel genome editing technologies, such as RNA interference (RNAi), zinc finger nucleases (ZFN) and plasmids, morpholinos, TALEN and CRISPR/Cas9 for functional analysis including targeted gene knockdown and knockout in various species including zebrafish, tilapia, and catfish [206,207,208,209,210,211,212,213,214,215,216,217,218]. Morpholinos, on the other hand, provide better specificity than RNAi (siRNA/shRNA/esiRNA) by decreasing the possibility of catastrophic off-target antisense effects [219], and has been widely used for studies in zebrafish and goldfish [217,218]. However, use of these technologies in catfish model has not been explored due to year long duration for development to maturation. Nevertheless, future studies need to be performed on this line to obtain novel information. In many animal models including catfish, RNA knockdown can be achieved more feasibly using siRNA, shRNA or esiRNA. In this line, in vivo and in vitro transient gene silencing using PEI mediated siRNA/shRNA/esiRNA has been standardized and well established at tissue and cellular levels in gonads and brain as well as at animal level in our laboratory using various fish models including catfish [67,85,136,165,188,220,221] to functionally characterize many important factors related to teleostean reproduction. In addition, Senthilkumaran [168] compared mammalian and piscine oocyte maturation with a note on sperm maturation citing the involvement of *hsd20b* vis-à-vis 17α,20β-DP in addition to T and 11-KT [140,222]. In line with these, more detailed knock-down analysis can be performed. Orchestration of various genes during different stages of gametogenesis/gonadogenesis of catfish has been schematically represented in the Figure 2.

## 7. Future Perspectives

Sex determining genes are the master switches controlling sex determination/differentiation in vertebrates including fishes. Catfishes have been used for decades now, to identify and characterize crucial genes and factors in reproduction and neuro-endocrine control of reproduction. Important findings from such studies have been summarized in the Table 1.

However, up to now, *Sry* and *dmy* have been the only sex-determining genes isolated in mammals and medaka [114,116], but neither *Sry* nor *dmy* homolog, other than *dmrt1* as testis-specific gene in autosomes, has ever been isolated in any other fish species, including catfish. However, Y-chromosome specific molecular markers have been identified using *sry*-specific PCR primers in cyprinid fish, *Puntius conchonius* [115]. Experimental evidence demonstrating *amh* function and other candidate genes in sex determination is less explored in catfish. Additionally, in the studies involving identification and characterization of steroidogenic enzyme genes using fish models, most of the time data stops at gene expression analysis through quantitative PCR. However, studies, from our laboratory, on localization, enzymatic assays and protein quantification indicated a robust way of analyzing the enzyme genes not only to distinguish tissue level activities but also seasonally [169]. As most of the catfish species do not spawn naturally under laboratory conditions, studies comparing GTH-induced models together with the use of advanced NGS techniques might leads to discovery/identification of crucial players in spawning and might provide new insights to understand its molecular mechanisms. This makes the use of seasonally breeding catfish unique and advantageous for such studies. Moreover, identification and characterization of novel sex determination related genes which are crucial to understand the masculinization/feminization mechanisms will help and promote aquaculture immensely across teleost including catfish.

## Figures and Tables

**Table 1:** Studies in catfish species: Identification of crucial genes/factors in reproduction and its neuroendocrine regulation.

Catfish Species	Nature of Study	Markers (Genes/Factors/Hormones) Studied	Highlights	References
*C. magur* (*C. batrachus*)	Neuroendocrine regulation	*th*	Female specific high expression of *th* in brain during early development.	[62]
		*th*	Sexual dimorphism in the hypophysiotropic *th*-positive neurons in the preoptic area associated with LH cells.	[72]
	Neuroendocrine-reproductive axis	GTH-II	Development of a heterologous radioimmunoassay for GTH-II and indication of a dynamic positive/negative feedback relationship between gonadal steroids and GTH-II.	[42]
		MAO	Estimation of MAO activity in gonads during different reproductive phases with a sudden decline after spawning.	[54]
		COMT	Changes in ovarian OE_2_,OE-2-H and COMT depicts stimulation of CE synthesis and degradation during GnRH-induced ovulation.	[68]
		NPY	NPY receptors are involved in the secretogogue effects of NPY on LH and GH cells in the pituitary similar to mammalian Y1 receptors.	[82]
	Promoter motif analysis	*sox3*, *hsd11b*	Sox3 binds to *hsd11b* promoter and transactivates to regulate male reproduction.	[191]
	Reproductive endocrinology	*cyp11a1*	Exposure of MT and EE_2_ during testicular development showed lower *cyp11a1* levels in the testis and brain indicating a certain feedback intervention.	[196]
		*nr2c1*	Expression during pre-spawning phase and localization of *nr2c1* transcripts in sperm/spermatids.	[187]
	Transient gene silencing	*wt1*, *ad4bp/sf-1*, *gata4*	Transient silencing of *wt1*-esiRNA downregulated *ad4bp/sf-1* and *gata4* expression, along with steroidogenic enzyme genes related to androgen production.	[188]
	Transient gene silencing, promoter motif analysis	*pax2*, *wnt4*, *wnt5*	Synchronous expression of *pax2* and *wnt5* during the ovarian development and recrudescence. *pax2* siRNA treatment reduced the expression of ovarian development like signaling molecules– wnt4/5. Transcriptional interaction of Pax2 on *wnt5*.	[167,220]
*C. gariepinus*	Neuroendocrine regulation	GTH	Purification of GTH, development and validation of a homologous radioimmunoassay for GTH.	[6]
		cGnRH-II, cfGnRH,	cGnRH-II is the more potent GTH-II secretagogue than cfGnRH.	[11]
		cfGnRH-R1, cfGnRH-R2	cfGnRH-R1 showed higher affinity than cfGnRH-R2 for cGnRH-II, cfGnRH.	[14]
		DA, GnRH, GTH, LH-RHa	DA inhibited GnRH- induced GTH release.	[64,92]
		*tph*, 5-HT	Male specific expression of *tph* in preoptic area of hypothalamus during early development.	[47]
		*gfrα-1*	Transient silencing of *gfrα-1*-siRNA downregulated brain specific genes and MPTP exposure indicated an interaction between GFRα-1 and DA-ergic system.	[67]
	Promoter motif analysis	*cyp19a1b*, *ftz-f1*, *foxl2*	Synchronous expression of *cyp19a1b*, *ftz-f1* and *foxl2* in the brain with high *ftz-f1 and foxl2* expressionin the female brain.	[98]
		CRE, cAMP, *hsd20b*	Identification of CRE in *hsd20b* promoter and its modulation by cAMP implicating its role in FOM.	[155]
	Reproductive endocrinology	*dmrt1a*, *dmrt1b*, *dmrt1c*, MT	Identification of multiple *dmrt1*s as testis-specific markers upon MT treatment.	[120]
		StAR	Elevation of StAR during hCG-induced oocyte maturation, in vitro and in vivo.	[194]
	Neuroendocrine-reproductive axis	cGnRH-II, GTH-II, cfGnRH	Increase in 11-KT after cGnRH-II and cfGnRH treatment in 24 and 39 week-old fish respectively.	[90]
		GTH	Castration resulted in increased plasma GTH levels, decreased GTH content in pituitary. T and androstenedione (aromatizable androgens) could abolish the castration-induced increase in plasma GTH and restored pituitary GTH content, however, non-aromatizable androgens could not.	[93]
		CAs, GnRH-I, E_2_, MT, 11-KT	Controlled release of sex steroids modulates GnRH and CAs activity dimorphically. Brain-related transcripts were elevated after estrogenization as compared to androgenization.	[103]
		*cyp19a1a*, *cyp19a1b*	*cyp19a1a* plays critical role during ovarian differentiation and demonstration of female specific expression of brain *cyp19a1b* during ontogeny.	[158]
	Transient gene silencing	NPY	Significant decrease in expression of ovary-related transcripts after NPY-esiRNA transient gene silencing indicating a role of NPY in ovary through cfGnRH-GTH axis.	[85]
		*c-kit*, 11-KT, T	Decrease in 11-KT and T levels upon *c-kit* esiRNA silencing.	[136]
		*sycp3*	*sycp3* -esiRNA transient gene silencing affected the expression level of various testis-related genes.	[189]
*H. fossilis*	Neuroendocrine regulation	GTH, DA, 5-HT, NE, CE, COMT	Preovulatory decrease in DA content with rise in 5-HT and NE levels.	[8]
		hfGnRH2, *kiss2*	Characterization of brain *kiss2* and hfGnRH2. Kiss2-GnRH2 signaling is involved in photo-thermal-mediated mechanisms controlling reproduction.	[17,20,21]
		GTH, DA, NE, A	5-HT, NE and A are stimulatory to GTH secretion. Hypothalamic 5-HT content and turnover were inhibited after pCPA and melatonin treatment but the content and turnover of CAs were not. However, α-MPT treatment decreased the content and turnover of DA, NE, and A.	[52]
		E_2_, GTH, MAO	Half-life analysis and turnover study of hypothalamic MAO. E_2_ exerts feedback regulation of GTH.	[58]
		DA, NE, A, VT	Physiological changes in VT is differentially regulated by CAs wherein DA inhibits and NE/A stimulates vasotocin (VT).	[61]
		GTH-II	Ovariectomy-induced rise in GTH-II was regulated by activation of hypothalamic serotonergic and suppression of dopaminergic mechanisms.	[66]
		*th*, E2, pKA, pKC, cAMP	E_2_ modulated the short-term activation of brain *th* activity differentially and *th* activity could be positively correlated with the annual reproductive cycle.	[70,71]
		GABA, GTH-II, E_2_	GABA regulates GTH-II secretion even when dopamine receptor function is inhibited.	[79]
	Neuroendocrine-reproductive axis	GTH, E_2_, NE(_2_)R	High NE(_2_)R levels in pituitary, followed by hypothalamus and telencephalon in all the seasons. Ovariectomy exerted a strong negative feedback on GTH secretion in the prespawning phase.	[43]
		5-HT, MAO	Day-night variations of 5-HT and MAO are photoperiod-dependent and are controlled during the gonadal preparatory phase of the annual reproductive cycle.	[51]
		5-HT, MAO	High hypothalamic activities of 5-HT and MAO during recrudescence and day-night variations during the early and mid-preparatory phase.	[55]
		E_2_, 5-HT, MAO	E_2_ modulates MAO activity and alters hypothalamic 5-HT in seasonally dependent manner.	[56]
		DA, NE, A, E_2_	E_2_-negative feedback acts on CA to modulate GTH secretion.	[57]
		COMT, E_2_	COMT content increased with progress of ovarian recrudescence in all the brain regions and declined after spawning. Mammalian GnRH analogue injection increased ovarian OE-2-H at 8 h and restored to control level after egg-stripping at 16 h whereas ovarian OE_2_ and COMT activity was significantly decreased at 8 h.	[68,69]
		VT, isotocin, E_2_, T, progesterone, hCG, PGF2α, PGE2	Immunocytochemical distribution of VT. Steroid hormones and hCG modulated brain and ovarian VT dynamics. Like hCG, VT had differential effects on ovarian steroidogenesis. VT induced FOM/ovulation through the VT receptors and activation of VT secretion and ovarian recrudescence by long photoperiod and high temperature.	[172,173,174,175,176,177]
		DA, NE, A, propranolol	NE modulated FOM through β-adrenergic mechanism, implicating a neural control of oocyte maturation/ovulation	[178]
	Reproductive endocrinology	E_2_, T, cortisol	T acted as a precursor for estrogen synthesis and cortisol enhanced estrogen-induced vitellogenin synthesis.	[171]
*I. punctatus*	Gene-editing	LH	LH gene editing and sterilization using ZFN technology	[216]
	Neuroendocrine-reproductive axis	ccLHR, ccFSHR	Characterization of ccLHR and ccFSHR. LH, a key regulator of periovulatory maturational events, and seasonal changes in ovarian expression of the ccFSHR (peaked at the onset of ovarian recrudescence and decreased prior to spawning).	[38,39]
	NGS	*amh*, *dmrt1*, *dmrta2*, *dmrt3a*,among others	Identification of male-biased genes.	[200]
		*gsdf*, *cxcl12*, *nanog*, *pou5f1*,among others	Identification of male-preferential genes, such as *gsdf*, *cxcl12*, as well as other cytokines mediating the development of the gonad into a testis.	[201]
*C. punctatus*	Neuroendocrine-reproductive axis	5-HT	pCPA injection decreased both the content and activity of 5-HT.	[48]
		5-HT, DA, NE	Melatonin administration caused diurnal variations in 5-HT content and turnover with no effect to indole treatment. Melatonin caused significant reduction of NE with no affect on DA.	[50]
*P. fulvidraco*	Gene editing-CRISPR/Cas9	*pfpdz1*	Male-specific expression during sex differentiation. Overexpression of *pfpdz1* using additive transgenesis initiated testicular differentiation whereas targeted inactivation of *pfpdz1* using CRISPR/Cas9 triggered ovarian differentiation.	[190]
	NGS	*hsd20b*, *sox9a*, *spags*, *fgfbp2*, *dmrt1*, *cyp17a*, *igfbpii*, among others	Identification of sex-related genes.	[204]
		*dmrt1*, *sox9a/b*, *cyp19b*, *wt1*, *amh*, *dax1*, *sf1*, *vasa*, *nanos*, among others	Identification of candidate genes for sex determination/differentiation.	[205]
*A. seemanni*	Neuroendocrine regulation	5-HT, *th*	Localization of 5-HT positive neurons in the pineal stalk.	[59]
*S. nigriventris*	Neuroendocrine regulation	5-HT, *th*	*th1*-expressing dopamine cells (unlike *th2*-expressing ones) do not co-localize with 5-HT.	[59]
*M. cavasius*	Neuroendocrine regulation	5-HT	Melatonin inhibited reproductive activity through modulation of serotonergic activity.	[60]
*M. wyckioides*	NGS	*amhr2*, *gnrh*, *gnrhr*, *cyp19a*, *igf1*, *igf2*, *taar*, *pcdh16*, *gcnt3*, among others	Identification of 19 differentially expressed genes in the pituitary, annotated to 32 signaling pathways related to gonad development.	[197]
*C. fuscus*	NGS	*cyp17a1*, *cyp11c1*, *hsd3b1*, *hsd17b1*, *hsd17b2*, *tgfβ2*, *tgfβ3*,among others	Identification of sex-related genes.	[198]
*S. asotus*	NGS	*amh*, *dmrt1*, *fgfrl1a*, *wnt5a*, *tab3*, *lmnl3*, among others	Identification and sex-specific expression of candidate genes.	[199]

## Data Availability

Not applicable.

## Abbreviations

MPTP	1-methyl-1,2,3,6-tetrahydropyridine
11-KT	11-ketotestosterone
17α,20β-DP	17α,20β-dihydroxy-4-pregnen-3-one
E_2_	17β-estradiol
5-HT	5-hydroxytryptamine
A	adrenaline
CA	catecholamine
COMT	catechol-O-methyltransferase
CE	catecholestrogens
DA	dopamine
EE_2_	ethynyl estradiol
FOM	final oocyte maturation
FSH	follicular stimulating hormone
GABA	γ-aminobutyric acid
GTH	gonadotropin
GnRH	gonadotropin-releasing hormone
hCG	human chorionic gonadotropin
HHG	hypothalamo-hypophyseal-gonadal
LH	luteinizing hormone
MT	methyltestosterone
MIH	maturation-inducing hormone
MAO	monoamine oxidase
NPY	neuropeptide Y
NGS	next generation sequencing
NE	norepinephrine
pCPA	para-chlorophenylalanine
RNAi	RNA interference
StAR	steroidogenic acute regulatory protein
siRNA	short interfering RNA
T	testosterone
TH	thyroid hormone
*tph*	tryptophan hydroxylase
*th*	tyrosine hydroxylase
VT	vasotocin
ZFN	zinc finger nucleases
